# Latitudinal Distributions and Controls of Bacterial Community Composition during the Summer of 2017 in Western Arctic Surface Waters (from the Bering Strait to the Chukchi Borderland)

**DOI:** 10.1038/s41598-019-53427-4

**Published:** 2019-11-14

**Authors:** Jiyoung Lee, Sung-Ho Kang, Eun Jin Yang, Alison M. Macdonald, Hyoung Min Joo, Junhyung Park, Kwangmin Kim, Gi Seop Lee, Ju-Hyoung Kim, Joo-Eun Yoon, Seong-Su Kim, Jae-Hyun Lim, Il-Nam Kim

**Affiliations:** 1 Marine Environment Research Division, National Institute of Fisheries Science, Busan, 46083 South Korea; 2 Korea Polar Research Institute, Incheon, 21990 South Korea; 3 Woods Hole Oceanographic Institution, MS 21, 266 Woods Hold Rd., Woods Hole, MA 02543 USA; 4 3BIGS, Hwaseong, 18454 South Korea; 5 Marine Bigdata Center, Korea Institute of Ocean Science and Technology, Busan, 49111 South Korea; 6 Faculty of Marine Applied Biosciences, Kunsan National University, Gunsan, 54150 South Korea; 7 Department of Marine Science, Incheon National University, Incheon, 22012 South Korea; 8 Fisheries Resources and Environmental Research Division, East Sea Fisheries Research Institute, National Institute of Fisheries Science, Gangneung, 25435 South Korea

**Keywords:** Marine biology, Marine chemistry

## Abstract

The western Arctic Ocean is experiencing some of the most rapid environmental changes in the Arctic. However, little is known about the microbial community response to these changes. Employing observations from the summer of 2017, this study investigated latitudinal variations in bacterial community composition in surface waters between the Bering Strait and Chukchi Borderland and the factors driving the changes. Results indicate three distinctive communities. Southern Chukchi bacterial communities are associated with nutrient rich conditions, including genera such as *Sulfitobacter*, whereas the northern Chukchi bacterial community is dominated by SAR clades, *Flavobacterium, Paraglaciecola*, and *Polaribacter* genera associated with low nutrients and sea ice conditions. The frontal region, located on the boundary between the southern and northern Chukchi, is a transition zone with intermediate physical and biogeochemical properties; however, bacterial communities differed markedly from those found to the north and south. In the transition zone, *Sphingomonas*, with as yet undetermined ecological characteristics, are relatively abundant. Latitudinal distributions in bacterial community composition are mainly attributed to physical and biogeochemical characteristics, suggesting that these communities are susceptible to Arctic environmental changes. These findings provide a foundation to improve understanding of bacterial community variations in response to a rapidly changing Arctic Ocean.

## Introduction

Arctic air temperatures have risen twice as fast as the global average (~0.7 °C) since the mid-20th century^1,2^. Recent Arctic warming is strongly linked to declining sea ice extent, suggesting a strong positive ice-temperature feedback^1,3^. As a result, annual sea ice extent has declined rapidly (3.5–4.1% per decade since 1979), which has resulted in physical and biogeochemical changes in the Arctic Ocean^2^. For instance, freshening of the upper Arctic Ocean enhances stratification and inhibits vertical mixing^2,4–6^, which in turn limits the transfer of deep ocean nutrients into the euphotic zone^7^. Ultimately, these physical changes will have a critical impact on Arctic primary productivity^8–12^.

**Figure 1:**
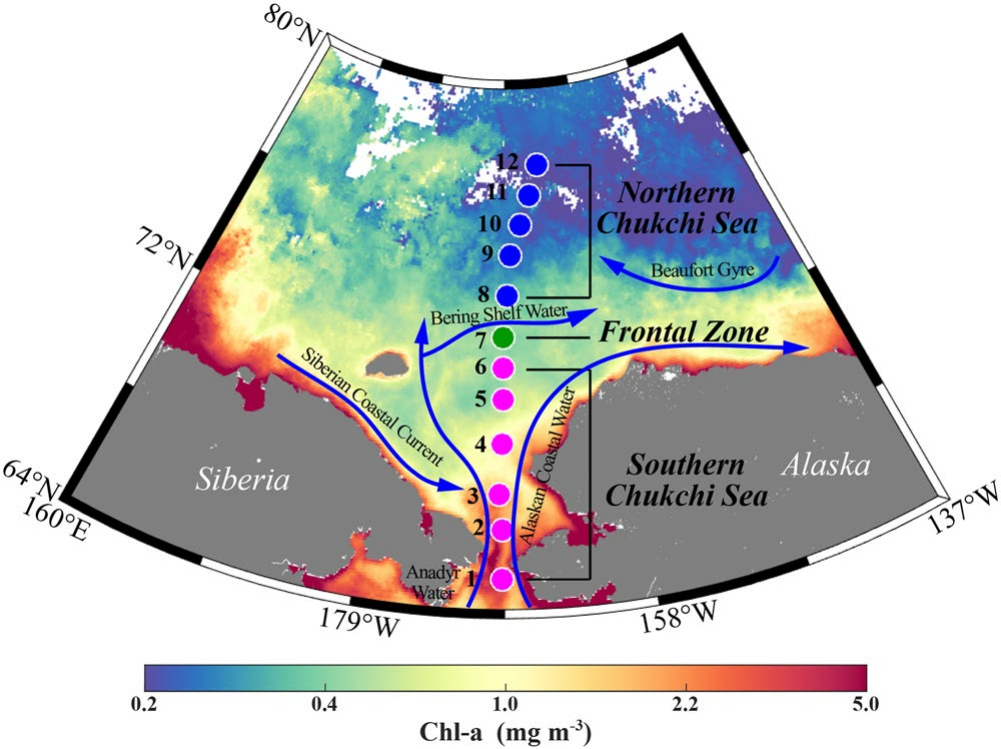
Map of the August 2017 Ice Breaking RV Araon western Arctic Ocean sampling stations used in this study. The location of each sampling site has been superimposed onto the Chl-a concentration contour (blue to red background colors). Pink, green, and blue circles represent stations in the Southern Chukchi (SC), Frontal Zone (FZ), and Northern Chukchi (NC) regions, respectively. Note that as satellite Chl-a data during August 2017 contained a large gap over the study area due to cloud/ice interferences, we show the mean state of summer Chl-a concentrations averaged from satellite Chl-a data obtained during August 2002–2017.

The Arctic Ocean is comprised of two distinct oceanic regions, distinguished by the presence (western Arctic) or absence (eastern Arctic) of Pacific water within the halocline. The western Arctic Ocean is geographically comprised of the Chukchi, East Siberian and Beaufort Seas, the Canadian Arctic Archipelago, and the Canadian Basin (Fig. 1)^13^. This ocean has experienced the most rapid sea ice retreat^14^, which has been driven by heat transport from Pacific waters^15–17^. Relatively warm, fresh, and nutrient-rich Pacific waters enter the western Arctic Ocean through the Bering Strait to the Chukchi Sea, which is a shallow (average depth 50 m) and wide (surface area 620 × 10^3^ km^2^) sea^18–20^. During the summer open water season, latitudinal differences in the physical and biogeochemical features of the western Arctic surface water are apparent from the Bering Strait to the Chukchi Borderland^5,21^. Relatively low latitude regions (i.e., the Bering Strait to the Chukchi Shelf) are primarily driven by Pacific waters that supply nutrients and heat, and are among the world’s most productive ocean regions^22–24^. Conversely, the higher latitude regions (i.e., Chukchi Borderland and Canada Basin) are relatively cold, fresh, and oligotrophic because the surface layer is highly influenced by freshwater inputs from melting ice and rivers through the Beaufort Gyre. Intermixing of the two surface water masses in the western Arctic has produced a physicochemical frontal zone in the Chukchi Sea^5^.

The latitudinal gradient of surface water physical and biogeochemical features in the western Arctic also affects the marine planktonic ecosystem^8,21,25–27^. Previous studies have shown that large-size phytoplankton groups and a high chlorophyll-a (Chl-a) biomass are associated with high nutrient Pacific waters in lower latitude regions, while small-size phytoplankton groups and a low Chl-a biomass are attributed to the oligotrophic conditions of higher latitude regions^12,21^. A latitudinal gradient of abundance and diversity in the summer zooplankton community of the western Arctic has also been reported^26^.

Bacterial communities are a key component of the marine ecosystem because they are responsible for modifying and decomposing organic matter, supporting higher trophic levels and driving biogeochemical cycles^28–32^. Recent studies have reported that the composition and relative abundance of bacterial communities in the western Arctic Ocean are largely determined by physical and biogeochemical water properties, such as temperature, salinity, and nutrients, indicating that microbial communities are highly susceptible to environmental changes^33–37^. However, little is known about changes in the latitudinal distribution of bacterial community compositions in response to western Arctic environmental changes^9,21,38^. Based on the studies cited above, we expect that future changes in the western Arctic Ocean will have a profound impact on the latitudinal gradient of physical and biogeochemical features and will in turn alter the Arctic marine ecosystem. To predict the response of western Arctic marine ecosystems to future changes, an investigation of the latitudinal patterns of bacterial community composition and their physical and biogeochemical properties is vital.

Here, we present (1) an investigation into the latitudinal distribution of physical-biogeochemical-bacterial community features in western Arctic surface waters during the summer open water season, and (2) a discussion of the environmental parameters governing the latitudinal distribution of the bacterial community. The goal is to provide significant insight into latitudinal gradients in marine ecosystem alterations in response to rapid Arctic climate change.

## Materials and Methods

### Sample collection

In August 2017, surface seawater (0–2 m) samples were collected from the Bering Strait to the Chukchi Borderland in the western Arctic Ocean at 12 stations occupied by the ice breaker RV Araon (Fig. 1). At each sampling location, approximately 3 L of seawater was collected using Niskin bottles attached to a conductivity, temperature, and depth (CTD) rosette sampler. For bacterial community analysis, 2 L of seawater was filtered through a 0.2 μm membrane (Whatman 47 mm polycarbonate membrane) to capture microbial cells. Filter samples were then immediately frozen and stored at ‒80 °C until DNA extraction. The 12 sampling locations were divided into three groups according to surface seawater properties: the southern Chukchi (hereafter SC, Stations 1–6), the frontal zone (hereafter FZ, Station 7), and the northern Chukchi (hereafter NC, Stations 8–12)^6,22,26^ (Fig. 1).

### Measurements of physical and biogeochemical parameters

Physical and biogeochemical parameters were measured at each station. The vertical profiles of temperature and salinity were measured using the CTD (Sea Bird 911plus, Electronics) and density was derived from the same. Nutrient samples (i.e., ammonium, nitrate + nitrite, phosphate (PO_4_), and silicate (SiO_2_)) were analyzed in the onboard laboratory using a continuous flow auto analyzer (QuAAtro, Seal Analytical, UK). In this study, dissolved inorganic nitrogen (DIN) represents the sum of ammonium, nitrite, and nitrate. Cells for Chl-a analysis were filtered onto 25 mm Whatman GF/F filters, extracted in 90% acetone at 4 °C for 24 hours, and quantified using a Turner Designs fluorometer (Trilogy Fluorometer, Turner Designs, USA).

### DNA isolation and sequencing of 16S rRNA

Total microbial community DNA was extracted from 0.2 μm filters using a standard protocol DNA isolation kit (Qiagen, Germany), and the quality of the extracted DNA was checked using NanoDrop (Thermo Scientific, USA). After performing quality control (QC), the samples were used for library preparation. The 16S rRNA genes were amplified using 16S V3-V4 primers. The primer sequences were as follows: 16S V3-V4 primer (16S Amplicon PCR Forward Primer 5′ TCGTCGGCAGCGTCAGATGTGTATAAGAGACAGCCTACGGGNGGCWGCAG) and (16S Amplicon PCR Reverse Primer 5′ GTCTCGTGGGCTCGGAGATGTGTATAAGAGACAGGACTACHVGGGTATCTAATCC). Polymerase chain reaction (PCR) products were purified, and a subsequent limited-cycle amplification step was performed to add the multiplexing indices used to construct the library. The extracted DNA samples were amplified and analyzed using llumina Miseq (Miseq Control Software v2.4.1.3) and Real Time Analysis (RTA v1.18.54.0). Then, 16S tagging DNA fragmentation (100‒500 bp) was performed using the quantitative kit for marine sample DNA. Primers were prepared for all DNA fragments, amplified using an adapter, and subjected to library QC using a bioanalyzer and clustering.

### Bioinformatics analysis

Sequencing data were processed using QIIME1.9.1 (https://qiime2.org) to assemble paired end reads into tags according to their overlapping relationship. In the pre-processing step, the primer was removed, and then demultiplexing and quality filtering (Phred ≥20) were applied^39^. USEARCH7 was used to perform denoising and chimera detection/filtering in operational taxonomic units (OTUs) grouping^40^. Then, the Silva132 database was used to determine the OTUs with 97% similarity using UCLUST and the open-reference analysis method, and determined the OTU identifier. OTU table was normalized dividing each OTU by the 16S copy number abundance, and in turn normalization of OTU table was performed. After filtering the generated OTU table using the Biological Observation Matrix (BIOM) format, the resulting sequences were clustered into OTUs based on a similarity threshold of ≥97% using Python Nearest Alignment Space Termination (PyNAST)^41^.

### Data visualization and statistical analysis

**Figure 2:**
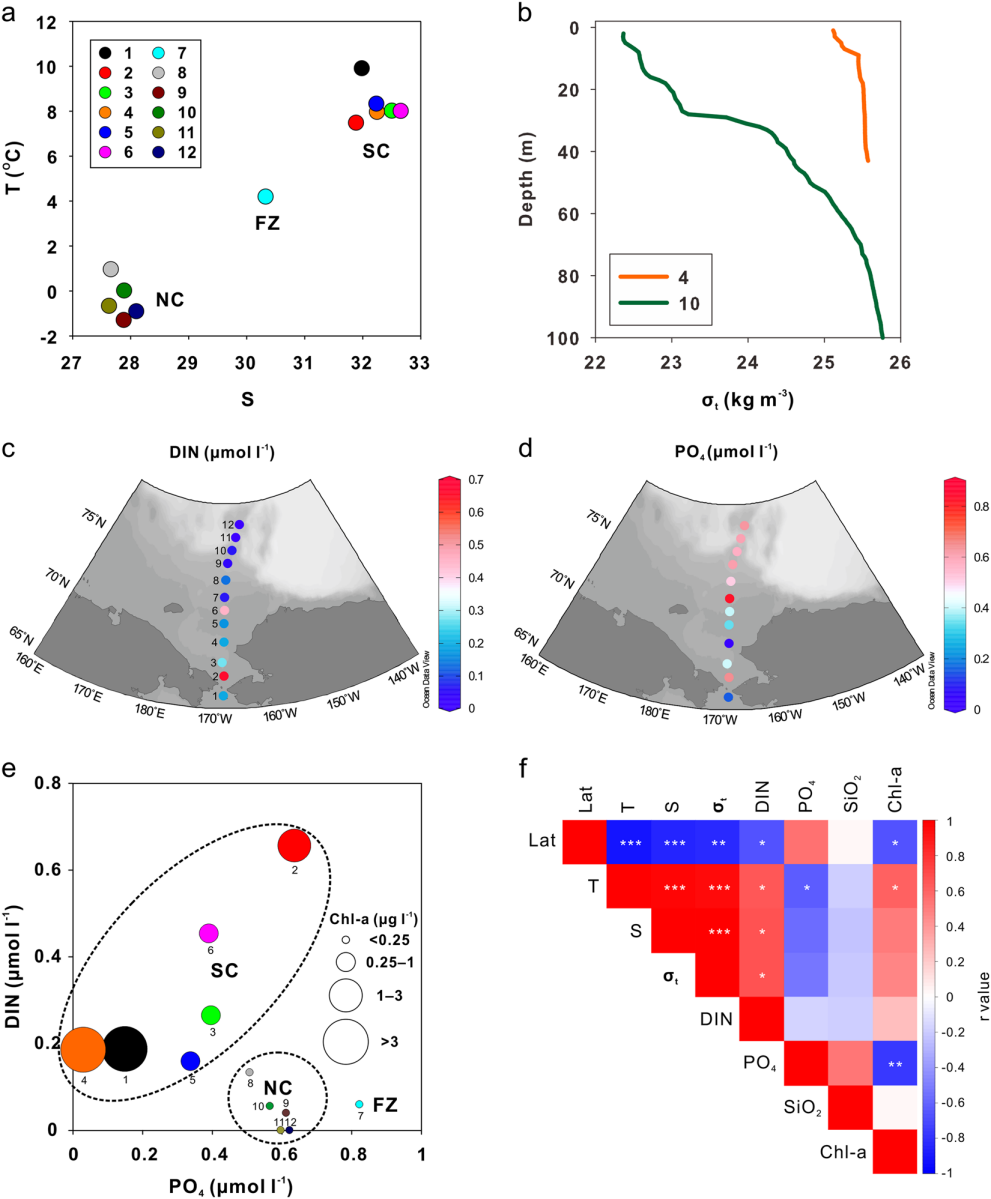
Physical and biogeochemical characteristics of the western Arctic Ocean: (**a**) temperature-salinity diagram of surface waters; (**b**) density (σ_t_) profiles at Stations 4 and 10, representative of the SC and NC, respectively; surface water distribution of (**c**) dissolved inorganic nitrogen (DIN) and (**d**) phosphate (PO_4_); (**e**) PO_4_ versus DIN in surface waters (bubbles indicate the Chl-a concentration); and (**f**) Pearson’s correlation coefficient matrix of physical and biogeochemical factors. The symbols of *, **, and ***indicate p value less than 0.05, 0.01, and 0.001, respectively.

**Figure 3:**
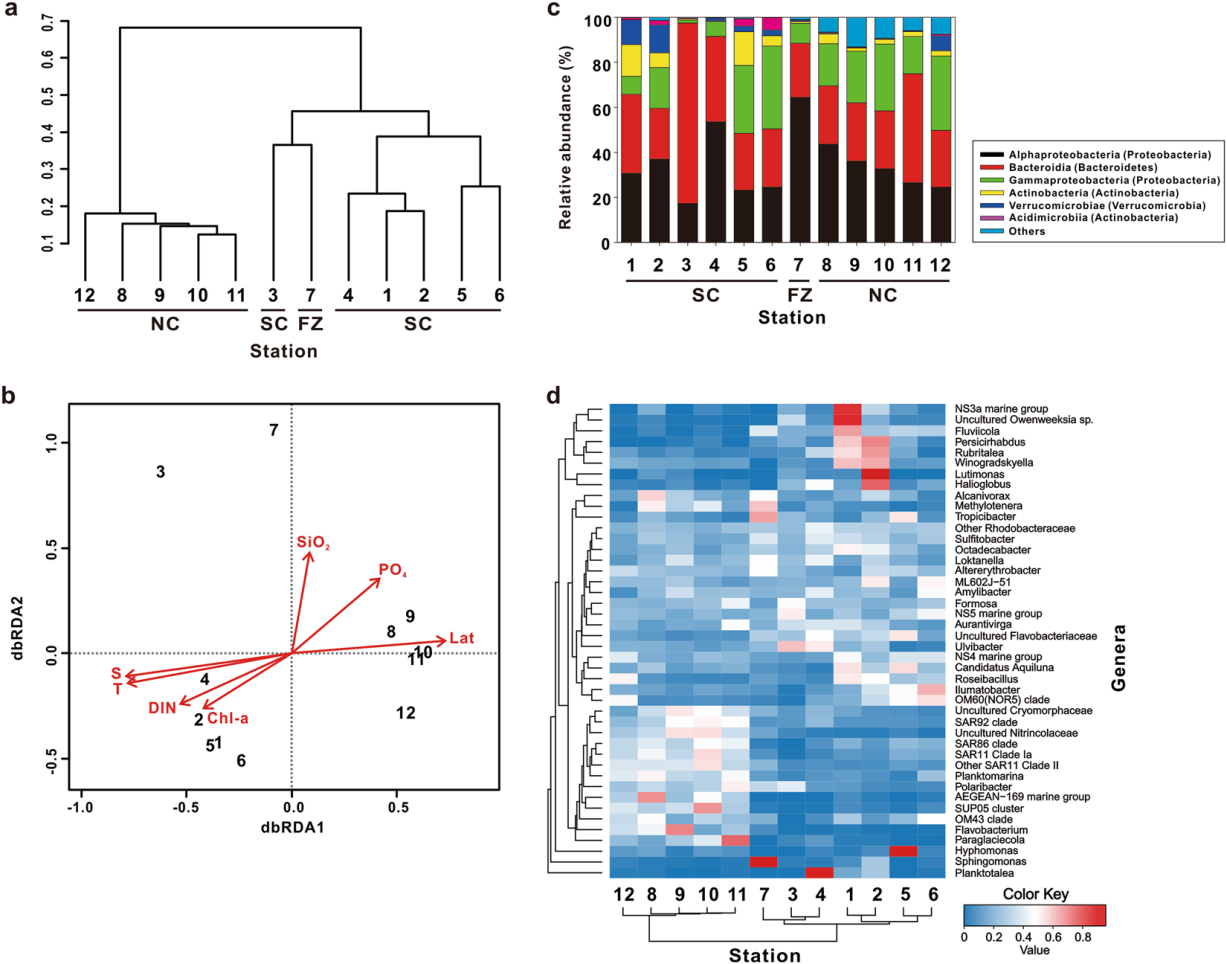
Western Arctic Ocean bacterial community characteristics: (**a**) clustering of samples at the operational taxonomic unit (OTU) level based on the Bray-Curtis distance (SC, FZ, and NC indicate the regional groups as defined in Fig. 1); (**b**) distance based redundancy analysis (dbRDA) ordination diagram of bacterial communities based on OTU with related physical and biogeochemical factors including latitude (Lat), temperature (T), salinity (S), dissolved inorganic nitrogen (DIN), phosphate (PO_4_), silicate (SiO_2_), and Chl-a, represented by arrows; (**c**) relative abundance of bacterial community composition at the class level; and (**d**) hierarchical heatmap showing the relative abundance at the genus level. Color code indicates the relative abundance, ranging from blue (low abundance) to red (high abundance). For (**c,d**) represented taxa occurred at >1% abundance in at least one sample.

**Figure 4:**
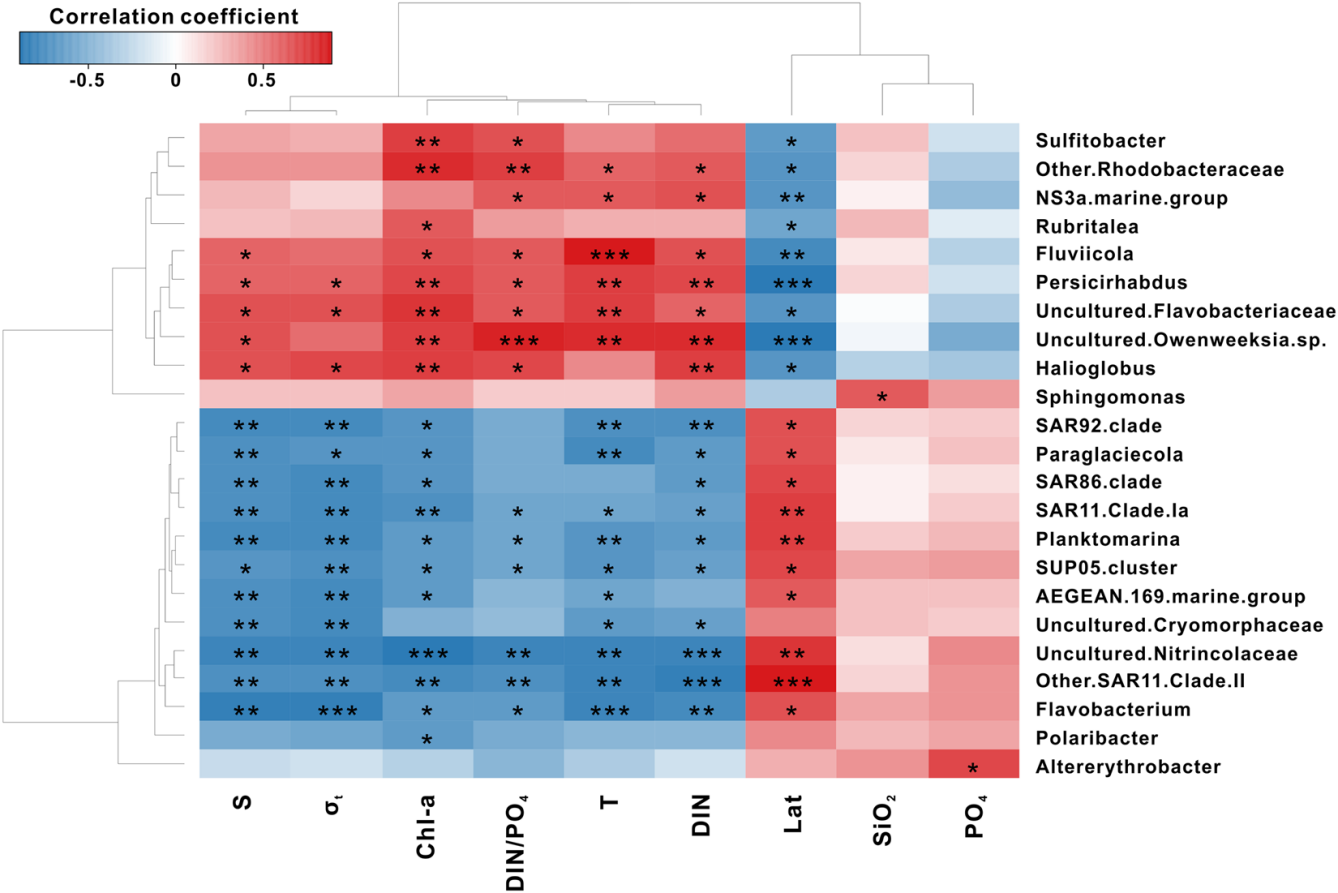
Hierarchical heatmap showing the Spearman’s Rank Order Correlation coefficient between the relative abundance of the bacterial community at the genus level (>1% abundance in at least one sample) and the physical and biogeochemical factors of Lat, T, S, σ_t_, DIN, PO_4_, DIN/PO_4_, SiO_2_, and Chl-a.

Pearson’s correlation coefficients (Fig. 2f) were calculated for normally distributed data including latitude, temperature, salinity, density, dissolved inorganic nitrogen (DIN), PO_4_, SiO_2_, and Chl-a using R software (v.3.5.1, https://www.R-project.org). A Hellinger-transformation was used for the statistical analysis of bacterial community relative abundance. For clustering analysis, the Bray-Curtis distance (Fig. 3a) was calculated to obtain diversity information using the R package vegan (ver. 2.5‒3). To analyze the relationship between microbial community composition and physical and biogeochemical parameters, a distance based Redundancy analysis (dbRDA) (Fig. 3b) was also performed using the R package vegan. Spearman’s rank order correlation coefficients (Fig. 4) were calculated for non-normally distributed data (i.e., between the relative abundance of bacterial taxa and physical and biogeochemical parameters) using R software. Additionally, the linearity based on the dbRDA, was evaluated using the Detrended Correspondence Analysis available in the R package vegan (refer Supplementary Text S1)^42^. Heatmaps (Figs 3d and 4) were generated using the R packages Heatplus (ver. 2.26.0), vegan, RColorBrewer (ver. 1.1‒2), and gplots (ver. 3.0.1). To determine whether the number of clusters shown in Fig. 3a can produce a significant result, the Caliǹski–Harabasz index^43^, a statistical estimate of the optimal number of clusters for clustering analysis, was estimated using the R package NbClust ver. 3.0 (refer the Supplementary Text S2), and in turn analysis of similarities (ANOSIM), which is a way to test statistically whether there is a significant difference between groups^44^, was conducted to determine cluster significance using the R package vegan.

## Results and Discussion

### Latitudinal physical and biogeochemical properties

Field observations during the summer of 2017 revealed strong physical and biogeochemical gradients from the Bering Strait to the Chukchi borderland (Fig. 2). Surface water masses were well defined according to their salinity and temperature in the western Arctic Ocean SC, FZ, and NC (Figs 1 and 2a). The mean temperature and salinity of surface waters were relatively high in the SC (8.3 °C and 32.2, respectively) compared to the NC (-0.4 °C and 27.8, respectively). Frontal features associated with mixing between the warm saline SC and cold fresh NC water masses were apparent at the FZ (Fig. 2a). In particular, mixing between freshwater inputs (ice melt and river discharge) from the north and inflow of relatively warm and saline Pacific waters from the Bering Strait forms a distinct physical gradient during the summer in the western Arctic Ocean^5,17,21^ as the NC is heavily impacted by summertime freshwater inputs that lead to development of a strong pycnocline and therefore an isolated surface water mass^4,5^ (Fig. 2b).

In August 2017, surface waters generally exhibited nutrient depletion: DIN < 0.66 μM, PO_4_ < 0.82 μM, and SiO_2_ < 4.62 μM (except at Station 7 where SiO_2_ = 9.40 μM). In particular, nitrogen (N)-deficiency (DIN:PO_4_ < 6.33) was prominent in surface waters (Table S1). Latitudinal differences in DIN, PO_4_, and Chl-a concentrations were also apparent between SC and NC (Fig. 2c–e). The mean DIN concentration was relatively high in the SC (0.32 μM) compared to the NC (0.05 μM), whereas the mean concentration of PO_4_ displayed the opposite pattern (i.e., 0.58 μM in NC > 0.33 μM in SC, Fig. 2c,d). Chl-a concentrations were markedly higher in the SC (2.61 μg l^−1^) compared to the NC (0.09 μg l^−1^, Fig. 2e and Table S1). The FZ displayed intermediate physical and biogeochemical characteristics (Fig. 2).

Correlation analysis among environmental variables (latitude, temperature, salinity, density, DIN, PO_4_, SiO_2_, and Chl-a, Fig. 2f) suggests significant latitudinal gradients, supporting the characterization of SC and NC waters as productive and oligotrophic, respectively. The SC’s productivity is influenced by input of nutrient-rich Anadyr waters that enter from the Bering Sea^12,21,45^, whereas the NC’s oligotrophy is due to the input of freshwater, leading to strong stratification that inhibits nutrient supply to the surface layer^22,46,47^. It has been reported that phytoplankton and zooplankton communities are in line with the physical and biogeochemical contrast between the SC (i.e., Arctic shelf regions) and the NC (i.e., Arctic deep basins)^5,12,21^.

### Latitudinal bacterial community structure

Caliǹski–Harabasz index^44^ (see Supplementary Text S2) cluster analysis based on the OTUs of the twelve surface water samples showed that bacterial communities were largely divided into three clusters of FZ plus Station 3, NC, and SC (excluding Station 3) with statistical significance (r = 0.86 and p < 0.001 from ANOSIM result) (Fig. 3a). According to dbRDA (Fig. 3b), bacterial community compositions are significantly linked to physical and biogeochemical factors (p < 0.05). In particular, SC communities are closely linked to temperature, salinity, Chl-a, and DIN, while NC communities are associated with PO_4_ and latitude (Fig. 3b). This result supports the suggestion that latitudinal changes in physical and biogeochemical characteristics play an important role in determining the distribution of these western Arctic bacterial communities^35,37,48–51^. That being said, the Station 3 and FZ communities, which are loosely linked to SiO_2,_ are virtually independent of physical and biogeochemical parameters (Fig. 3b).

To characterize the differences in bacterial community compositions with latitude, the relative abundance (%) of bacterial communities at the class level was analyzed (Fig. 3c). Alphaproteobacteria (mean 35%), Bacteroidia (mean 34%), and Gammaproteobacteria (mean 19%) were the dominant classes, accounting for 87% of all OTUs. Other major classes consisted of Actinobacteria (mean 5%), Verrucomicrobiae (mean 3%), and Acidimicrobiia (mean 1%). Overall, the bacterial community composition found in this study was consistent with those of previous studies in the western Arctic Ocean^35–37,48,49,52,53^. The relative abundance of Proteobacteria, including Alphaproteobacteria and Gammaproteobacteria showed an increasing tendency with latitude (Fig. S1). At the FZ, Alphaproteobacteria was the dominant class (a relative abundance of >60%), while Bacteroidia was the dominant class at Station 3 (a relative abundance of ~80%). Differences in dominant phylogenetic bacterial taxa at the genus level were investigated (Fig. 3d). Overall, the result of hierarchical clustering analysis was similar to Fig. 3a. The NC group showed a strong clustering association, whereas the SC and FZ groups were relatively loose. Interestingly, the relative abundance of *Sulfitobacter* (of the family Rhodobacteraceae) was from 4–29% (the relative abundance of Rhodobacteraceae: 11–52%) throughout the study area but the relative abundance was the highest in the eutrophic conditions of the SC (Fig. 2c‒e and Table S2). An earlier pyrosequencing study conducted in the Chukchi Borderland also reported presence of Rhodobacteraceae including *Sulfitobacter* with the relative abundance of 29–39%^37^. *Sulfitobacter* have been found to play an important role in the production of dimethyl sulfide (DMS), a gas with a cooling effect on the Earth’s climate^37,54,55^, through the assimilation of dimethylsulfoniopropionate (DMSP) released by the decomposition of organic matter in the water column after a phytoplankton bloom^56,57^. *Fluviicola* and *Persicirhabdus*, which are known to favor organic matter enriched conditions^53^, were also found in the SC.

Several taxa, including SAR (i.e., SAR11 clade Ia and other SAR11 clade II), SAR86 clade, SAR92 clade, *Flavobacterium*, and *Polaribacter* were relatively more abundant in the NC (Fig. 3d). SAR is known to dominate in oligotrophic environments^58–60^ and we found, in agreement with previous studies (Table S2)^36,37,49,53^, that SAR was more widely distributed in the oligotrophic NC (6–15%) as compared to SC and the FZ (<~2%).

*Polaribacter* (of the class Bacteroidia) was one of the dominant groups in the study area. However, the relative abundance was higher in the NC (10 ± 6%) than in the SC and FZ (4 ± 5%) (Table S2). In addition, the relative abundance of SAR92 clade (of the class Gammaproteobacteria) was higher in the NC (1–5%) than in the SC and FZ (0–1%) (Table S2). These results are consistent with earlier Chukchi Borderland surface waters findings^35,37^. *Sphingomonas*, a member of the class Alphaproteobacteria, was relatively abundant in the FZ (~6%) (Table S2). As the ecological characteristics of *Sphingomonas* are not yet well known, we could not determine whether frontal mixing, leading to a large gradient in physical and biogeochemical properties (Fig. 2), formed this unique bacterial community composition as an environmental adaptation. Alternatively, *Sphingomonas* may be representative of bacterial taxa intrusions from the subsurface to the surface via vertical mixing^48^. Subsurface bacterial community composition data were not available in this study, however these results suggest that future investigations should focus on the bacterial community composition of the entire water column to fully understand their microbial and biogeochemical roles in the study area^35,48^.

### Environmental factors determining latitudinal bacterial community composition

To investigate the influence of environmental factors on latitudinal bacterial community distribution we conducted a correlation analysis between the relative abundances of the top 23 genera and environmental parameters (latitude, temperature, salinity, density, DIN, PO_4_, DIN:PO_4_, SiO_2_, and Chl-a, Fig. 4).

Dominant genera in the SC are *Rubritalea*, *Sulfitobacter*, NS3a marine group, uncultured *Flavobacteriaceae*, *Fluviicola*, and *Persicirhabdus*, which show significant positive correlations with temperature, salinity, density, DIN, DIN:PO_4_, or Chl-a. These relationships suggest that SC bacterial compositions are influenced by the variability of water-column stability and nutrient conditions^61^. Dominant genera in the NC are SAR clades (SAR11 clade Ia and other SAR11 clade II), SAR86 clades, *Paraglaciecola*, AEGEAN 169 marine group, *Flavobacterium*, *Polaribacter*, *Planktomarina*, and SAR92 clades, which show mostly negative correlations with environmental variables. As found by earlier studies^36,37^, oligotrophic-type SAR clades were dominant in NC surface waters due to N-deficient conditions (Fig. 2). Furthermore, *Flavobacterium, Paraglaciecola, and Polaribacter* associated with sea-ice algal aggregates were found in the NC, indicating that sea-ice melting is an important driver of NC bacterial composition^62^.

Dominant genera in the FZ are *Sphingomonas* and *Altererythrobacter*, accounting for a relative abundance of approximately 6‒11% (Fig. 4). *Sphingomonas* has a significant positive correlation with SiO_2_ (r = 0.6, p < 0.05), while other *Altererythrobacter* are positively correlated with PO_4_ (r = 0.7, p < 0.05). Overall, FZ bacterial compositions differ markedly as compared to those of SC and NC, despite the intermediate physical and biogeochemical characteristics of the region (Fig. 2). We speculate that this difference could be attributed to: (1) an upward extension of subsurface bacterial communities due to vertical mixing^49^ or (2) frontal mixing forming unique bacterial communities adapted to an environment with large gradients in physical and biogeochemical properties. These speculations require further examination to improve our understanding of changes in bacterial community composition in the rapidly changing Arctic.

### Two potential scenarios of changes in bacterial community composition in response to rapid Arctic changes

Rapid environmental changes in the Arctic Ocean may influence Arctic bacterial community compositions both directly and indirectly. Among the changes observed in the region, two phenomena have received considerable attention; increasing inflow of warm Pacific waters^15–17^ and increasing sea-ice melt water^7,34^. Based on the results of this study, we can derive potential scenarios as to how the bacterial community compositions revealed in this study will be altered by these phenomena in the western Arctic Ocean in the future.

**Figure 5:**
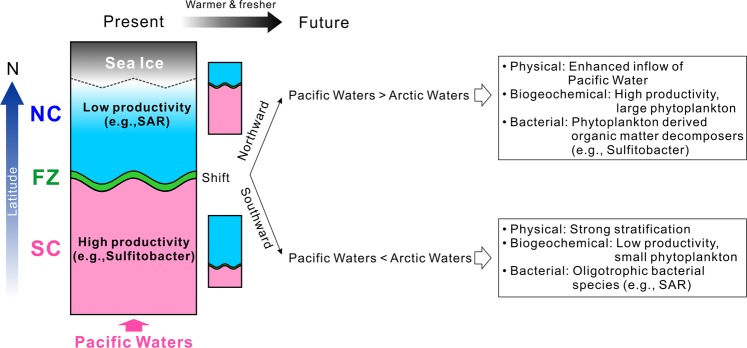
Schematic diagram of bacterial community distribution of western Arctic surface waters in response to future Arctic climate change.

If the impact of warm and nutrient-rich Pacific water inflows dominates^63,64^, it is likely that the productive SC region will expand and the FZ will move northward, leading to nutrient enrichment in the western Arctic (Fig. 5). In response, we expect that bacterial communities would be dominated by organic matter decomposers, such as *Sulfitobacter*, due to high primary productivity and phytoplankton of a larger size^12,21,25,26^. However, if the impact of sea-ice melt water dominates then the oligotrophic NC region will expand and the FZ will move southward, leading to nutrient depletion in western Arctic surface waters (Fig. 5). In this case, bacterial communities would be dominated by genera adapted to oligotrophic conditions, such as SAR clades^21,25,65^, and phytoplankton of a reduced size^12,65^. A recent study reported that nitrogen fixation by diazotrophs made a significant contribution to the nitrogen cycle in the western Arctic^66^. Also, the biogeochemical features shown in the NC surface waters (i.e., extremely low DIN and high PO_4_) are likely to have a potential for supporting the occurrence of nitrogen fixation (Fig. 2c‒f). However, nitrogen fixing bacteria were not identified in our study. The expansion of N deficient waters in the NC may stimulate the production of nitrogen in the surface layer via nitrogen fixation processes, suggesting that diazotrophs may become a dominant bacterial community in the future western Arctic.

## Summary and Conclusions

The latitudinal distribution of physical-biogeochemical-bacterial community features in western Arctic surface waters during summer 2017 was investigated. Results indicated that surface water bacterial communities were influenced by physical and biogeochemical characteristics in the western Arctic Ocean. We identified three distinctive groups associated with different environmental variables in the surface waters of the study area. The SC was relatively warm, saline, and productive, and its bacterial communities were dominated by phytoplankton-derived decomposers such as *Sulfitobacter*. The NC was characterized by extremely oligotrophic surface waters and strong stratification due to the influence of sea-ice melt with bacterial communities dominated by SAR clades, *Flavobacterium*, *Paraglaciecola*, and *Polaribacter*. The FZ was located at the boundary between the SC and NC and represented a transition zone with intermediate physical and biogeochemical properties. However, bacterial communities differed markedly as compared to both the SC and NC. *Sphingomonas*, whose ecological characteristics are not yet known, were relatively abundant. Results from the present study did not allow us to determine whether the unique bacterial community in the FZ can be attributed to an environmental adaptation or an extension of the subsurface bacterial community. We look to future investigations that would include sampling for microbial community composition over the entire water column and in other regions of the western Arctic to answer this question. Results from this study will aid in our understanding of bacterial community alterations in response to a rapidly changing Arctic Ocean.

## Supplementary information

Supplementary Information

Supplementary Table S2

## Data Availability

The raw sequence data have been deposited in the National Center for Biotechnology Information Sequence Read Archive (SRA) and are accessible through SRA accession number SRP159594.
