# Supplementary material for: Latitudinal Distributions and Controls of Bacterial Community Composition during the Summer of 2017 in Western Arctic Surface Waters (from the Bering Strait to the Chukchi Borderland)
Source: Sci Rep. 2019 Nov 14;9:16822. doi: 10.1038/s41598-019-53427-4 (PMC6856522; doi:10.1038/s41598-019-53427-4)
Supplement: Supplementary file 1 — Supplementary Information [file 41598_2019_53427_MOESM1_ESM.pdf]

-Supplementary Information-

Latitudinal Distributions and Controls of Bacterial Community Composition during the Summer of 2017 in Western Arctic Surface Waters (from the Bering Strait to the Chukchi Borderland)

Jiyoung Lee[1], Sung-Ho Kang[2], Eun-Jin Yang[2], Alison M. Macdonald[3], Hyoung-Min Joo[2], Junhyung Park[4], Kwangmin Kim[4], Gi Seop Lee[5], Ju-Hyoung Kim[6], Joo-Eun Yoon[7], Seong-Su Kim[7], Jae-Hyun Lim[8], and Il-Nam Kim[7*]

[1]Marine Environment Research Division, National Institute of Fisheries Science, Busan 46083, South Korea.

[2]Korea Polar Research Institute, Incheon 21990, South Korea.

[3]Woods Hole Oceanographic Institution, MS 21, 266 Woods Hold Rd., Woods Hole, MA 02543, USA.

[4]3BIGS, Hwaseong 18454, South Korea.

[5]Marine Big Data Center, Korea Institute of Ocean Science and Technology, Busan 49111, South Korea.

[6]Faculty of Marine Applied Biosciences, Kunsan National University, Gunsan 54150, South Korea.

[7]Department of Marine Science, Incheon National University, Incheon 22012, South Korea.

[8]Fisheries Resources and Environmental Research Division, East Sea Fisheries Research Institute, National Institute of Fisheries Science, Gangneung 25435, South Korea.

[*]Corresponding author: Il-Nam Kim (ilnamkim@inu.ac.kr)

Supplementary Materials: Text S1–S2, Table S1–[†]S2, and Figure S1.

[†]Table S2 is separately provided as Table S2.xls.

Text S1. Detrended Correspondence Analysis. This study uses redundancy analysis (dbRDA) to analyze the relationship between microbial community composition and physical and biogeochemical parameters. This technique assumes linearity. To test whether or not this assumption is valid for the data set used, the Detrended Correspondence Analysis (DCA) R package vegan ver. 2.5–3 was employed. This software calculates the DCA axis length. When the axis length is less than 3 standard deviations (SD) the linearity assumption is valid and only when it is longer is a unimodal method necessary. The results from the DCA software run for our data set are shown below and indicate that using a linear technique is adequate.

DCA axis	DCA1	DCA2	DCA3	DCA4
length	1.39	1.43	0.85	0.88

Text S2. Calinski-Harabasz index. To determine whether the number of clusters in Fig. 3a is significant, we used the Caliǹski–Harabasz (CH) index [S1], which provides the optimal number for clustering analysis and is given as:

$$CH = \frac{BCSM}{k-1} \times \frac{n-k}{\mathrm{WCSM}},$$

where *n* is the number of samples, *k* is the number of clusters, BCSM and WCSM are between-cluster variance and within-cluster variance, respectively. The BCSM is defined as:

$$\mathrm{BCSM} = \sum_{i=1}^{k} n_i \|m_i - m\|^2,$$

where n_i is the number of samples in cluster *i*, m_i is the centroid of cluster *i*, m is the overall mean of the sample data, and $\|m_i - m\|$ is the Euclidean distance between the two vectors. The WCSM is defined as:

$$\mathrm{WCSM} = \sum_{i=1}^{k} \sum_{x \epsilon c_i} \|x - m_i\|^2,$$

where *x* is a data point, and c_i is the *i*th cluster.

CH index was estimated using the R package NbClust ver. 3.0. As shown in the result below, the optimal number of clusters is 3.

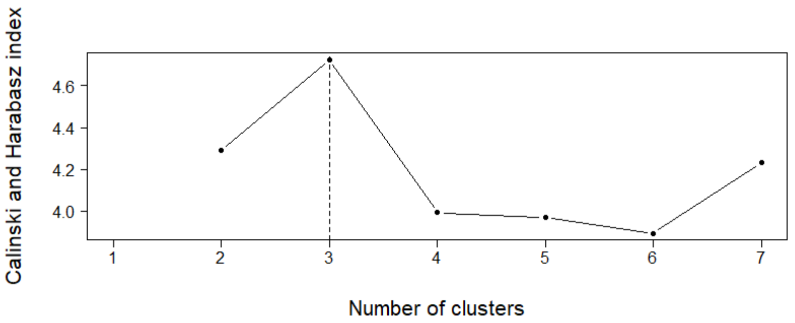

- Reference -

S1. Caliǹski, T.& Harabasz, J. A dendrite method for cluster analysis. Communications in Statistics, 3, 1–27 (1974).

Table S1. Physical and biogeochemical properties of surface water in 12 samples of western Arctic Ocean.

Station	Temperature	Salinity	Density	DIN	PO_4	Si	DIN/PO_4	Chl-a
1	9.9	32.0	24.6	0.19	0.15	4.05	1.26	5.82
2	7.5	31.9	24.9	0.66	0.64	4.62	1.03	2.25
3	8.0	32.5	25.3	0.27	0.40	2.05	0.67	0.42
4	8.0	32.2	25.1	0.19	0.03	2.70	6.33	6.10
5	8.3	32.2	25.1	0.16	0.34	0.00	0.47	0.49
6	8.0	32.7	25.4	0.45	0.39	0.00	1.16	0.57
7	4.2	30.3	24.1	0.06	0.82	9.40	0.07	0.25
8	1.0	27.7	22.1	0.13	0.51	3.10	0.26	0.11
9	-1.3	27.9	22.4	0.04	0.61	2.82	0.07	0.18
10	0.0	27.9	22.4	0.06	0.57	3.36	0.10	0.05
11	-0.7	27.6	22.2	0.00	0.60	2.97	0.00	0.04
12	-0.9	28.1	22.6	0.00	0.62	3.63	0.00	0.07

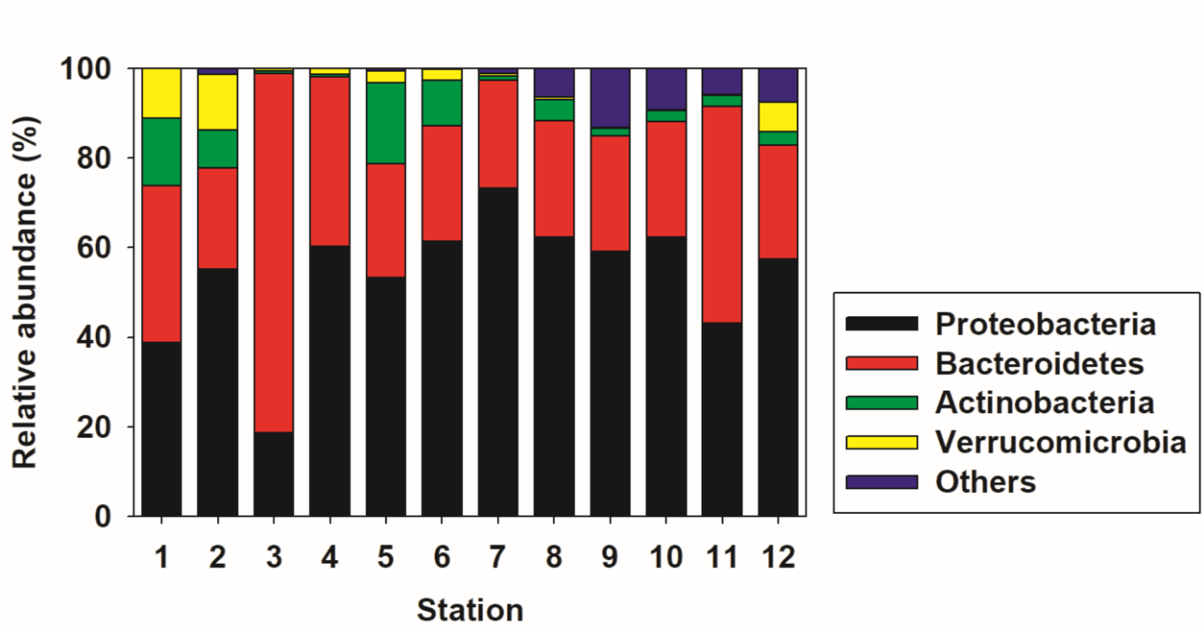

Figure S1. Relative abundance of bacterial community composition at the phylum level.
